# Development of a core set of outcome measures to be applied toward breast cancer-related lymphedema core outcome domains

**DOI:** 10.1007/s10549-024-07298-7

**Published:** 2024-03-22

**Authors:** David Doubblestein, Linda Koehler, Elizabeth Anderson, Nicole Scheiman, Paula Stewart, Mark Schaverien, Jane Armer

**Affiliations:** 1 Department of Physical Therapy, A.T. Still University, Mesa, AZ USA; 2 Department of Rehabilitation Medicine, University of Minnesota, Minneapolis, MN USA; 3 Sinclair School of Nursing, University of Missouri, Columbia, MO USA; 4 Occupational Therapy Assistant Program, Huntington University, Huntington, IN USA; 5 Parkridge Medical Center – Wound Care/Lymphedema Clinic, Parkridge Medical Center, Chattanooga, TN USA; 6 Division of Surgery, Department of Plastic Surgery, The University of Texas, MD Anderson Cancer Center, Houston, TX USA; 7 Professor Emerita, Sinclair School of Nursing, University of Missouri, Columbia, MO USA

**Keywords:** Breast cancer-related lymphedema, Core outcome set, Outcome measures, Tools, Instruments

## Abstract

**Purpose:**

For breast cancer survivors (BCS) living with breast cancer-related lymphedema (BCRL), what outcome measures (OMs) are recommended to be used to measure standardized outcome domains to fully assess the burden of the disease and efficacy of interventions? An integral component of a standardized core outcome set (COS) are the OMs used to measure the COS.

**Methods:**

A supplemental online survey was linked to a Delphi study investigating a COS for BCRL. OMs were limited to a maximum of 10 options for each outcome domain (OD). There were 14 ODs corresponding to the International Classification of Functioning, Disability, and Health (ICF) framework and respondents rated the OMs with a Likert level of recommendation. The feasibility of the listed OMs was also investigated for most outpatient, inpatient, and research settings.

**Results:**

This study identified 27 standardized OMs with a few ODs having 2–3 highly recommended OMs for proper measurement. A few of the recommended OMs have limitations with reliability due to being semi-quantitative measures requiring the interpretation of the rater.

**Conclusion:**

Narrowing the choices of OMs to 27 highly recommended by BCRL experts may reduce selective reporting, inconsistency in clinical use, and variability of reporting across interdisciplinary healthcare fields which manage or research BCRL. There is a need for valid, reliable, and feasible OMs that measure tissue consistency. Measures of upper extremity activity and motor control need further research in the BCS with BCRL population.

**Supplementary Information:**

The online version contains supplementary material available at 10.1007/s10549-024-07298-7.

## Background

**Fig. 1:**
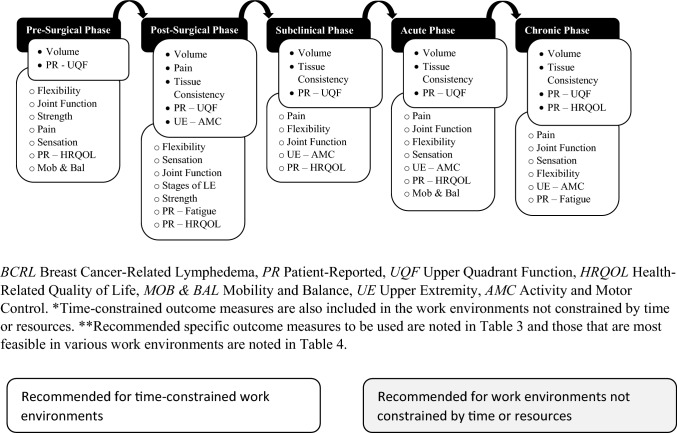
Recommended outcome domains to assess for each phase of the BCRL continuum

Standardized outcome measures (OMs) are incorporated into the examination of individuals with breast cancer-related lymphedema (BCRL) and are an essential component of evidence-based practice. OMs provide an outcome assessment of interventions for related impairments of body functions and structures, and limitations of activities and participation which can support clinical reasoning in the management of BCRL [1–3]. Recommended outcome domains (ODs) to be measured in breast cancer survivors (BCS) with BCRL were established through a Delphi study as a Core Outcome Set (COS) to be used in time-constrained clinic or research environments and where resources and time are not constrained [4]. The COS recommendations for time-constrained clinic and research environments included stages of lymphedema, volume, tissue consistency, pain, strength, patient-reported upper quadrant function, patient-reported health-related quality of life (HRQOL), upper extremity activity and motor control, and mobility and balance. The COS recommendations for clinic and research environments not constrained by resources or time included stages of lymphedema, volume, tissue consistency, body composition, joint function, flexibility, sensation, pain, strength, patient-reported fatigue, patient-reported upper quadrant function, patient-reported HRQOL, upper extremity activity and motor control, and mobility and balance. COS recommendations for the COS varied depending on the BCRL continuum phase (Fig. 1) [4].

Unfortunately, the use of OMs across a multidisciplinary group of professionals has been meager [5–7]. Professionals that either directly manage or research lymphedema include physical therapists (PTs), occupational therapists (OTs), massage therapists (MTs), nurses (RNs), and physicians (MDs) [5]. Lack of knowledge and competence to use OMs that measure ODs are routinely reported as significant barriers for the use of OMs across healthcare disciplines [8–10]. Other significant barriers to the use of OMs reported across disciplines include lack of time to implement, challenges with scoring and interpreting patient-reported OMs, difficulty in patient comprehension of patient-reported OMs, and lack of appropriate psychometric properties of the OMs [8, 10–12].

Doubblestein et al. [4] investigated 92 OMs that certified lymphedema therapists (CLTs) may use with BCS living with BCRL [13]. The authors discovered that 95% of CLTs most often used active range of motion, manual muscle testing (MMT), circumference measurements converted to volume, sensation, and manual tissue consistency measures when assessing BCRL [13]. However, these measures alone limit a specialist’s comprehensive understanding of the burden of this chronic condition and the identification of related co-morbidities that require specific interventions. CLTs have reported difficulty knowing the best OM to use due to numerous options [14]. Extensive choices of OMs that have acceptable psychometrics should be narrowed to guide healthcare professionals and researchers alike to gather best measures on ODs. Identifying OMs with good psychometric properties for the examination of BCS with BCRL benefits the patient and the professional, regardless of background (e.g. PT, OT, MT, RN, or MD). The foundation to identify recommended OMs has been laid by the Oncology Evaluation Database to Guide Effectiveness (EDGE) Task Force in identifying ODs and associated OMs with good psychometrics and clinical utility [15–18], the identification of OMs most often used by CLTs [13], and the development of a COS in different clinic and research environments [4]. The purpose of this study was to (1) develop a core set of standardized OMs to measure various ODs of BCRL, and (2) determine the feasibility of the recommended BCRL OMs for both clinic and research settings according to expert respondent viewpoints.

## Materials and methods

### Design

A supplemental survey (Supplemental Information A) was linked to a Delphi study investigating a COS for BCRL [4]. A Study Management Group (SMG) [19] was formulated by the principal investigator (DD), which included a multidisciplinary group of CLTs experienced with BCRL, including PTs (DD and LK), RNs (JA and EA), and an OT (NS). To further the representation of primary stakeholders of BCRL, a Study Advisory Group (SAG) was formed to provide additional expertise on the study development and data interpretation, which included a physiatrist (PS) and a microsurgeon (MS).

The supplemental survey was constructed through Qualtrics Software, Version January 2023 for internet dissemination by email. The surveys were developed by the SMG confirming face validity. The survey was piloted to the SAG substantiating content validity of the surveys. Content validation through expert judgement provides an informed opinion from qualified experts about how well a survey captures all relevant parts it aims to measure [20]. Forty-five of the 54 OMs investigated and included in the survey were selected based on a study that investigated the most often used OMs that CLTs incorporated in their assessment of BCS with BCRL (Table 1) [13]. To reduce survey burden, OMs with highest levels of use were included and options were limited to a maximum of 10 for each OD. Respondents rated the OMs as (1) highly recommended, (2) not as highly recommended, (3) not recommended, or (4) unfamiliar with the OM. There were 14 ODs corresponding to the International Classification of Functioning, Disability, and Health (ICF) framework. The ODs included under the ICF domain of body structures and functions were (1) progression of lymphedema, (2) joint function, (3) flexibility, (4) strength, (5) volume, (6) pain, (7) sensation, (8) tissue consistency, and (9) body composition. The ODs included under the ICF domain of activities and participation were (1) patient-reported HRQOL, (2) patient-reported upper quadrant function, (3) fatigue, (4) mobility and balance, (5) upper extremity and motor control. Hi-tech OMs were assessed separately which included (1) tissue water content and volume and (2) tissue consistency subdomains of ICF body structures and functions. The second component of the supplemental survey was to investigate the feasibility of the listed OMs. Respondents would rate each OM as (1) feasible in most outpatient settings, (2) feasible in most inpatient settings, (3) feasible in most research settings, or (4) no experience. See Supplemental Information B for OM descriptions.

**Table 1:** Outcome measures most often used (*n* = 111)

Outcome measure	Frequency of use *n* (%)
Circumferential measurements	108 (97.3%)
Pitting edema test—Palpation	108 (97.3%)
Tissue texture—Palpation	107 (96.4%)
Goniometer—active range of motion	107 (96.4%)
Manual muscle test	107 (96.4%)
Numeric pain scale	104 (93.7%)
Stiff glenohumeral joint	96 (86.5%)
Light touch brushing	92 (82.9%)
Goniometer—passive range of motion	91 (82.0%)
Body weight	89 (80.2%)
Lymphedema life impact scale	82 (73.9%)
Body mass index	81 (73.0%)
*Quick*DASH	80 (72.1%)
Visual analog scale—pain	78 (70.3%)
Hand grip dynamometer	69 (62.2%)
Pectoralis major length	68 (61.3%)
DASH	66 (59.5%)
Timed up and go	66 (59.5%)
Pectoralis minor length	64 (57.7%)
Berg balance scale	56 (50.5%)
Dynamic motion of scapula	54 (48.6%)
Sharp-dull discrimination	50 (45.0%)
Visual analog scale–fatigue	49 (44.1%)
Hand held dynamometry	47 (42.3%)
Five times sit to stand	43 (38.7%)
Six minute walk test	40 (36.0%)
Pinch dynamometer	38 (34.2%)
Functional reach	35 (31.5%)
Two-point discrimination	35 (31.5%)
Monofilament	35 (31.5%)
Brief fatigue inventory	27 (24.3%)
Shoulder pain and disability index	25 (22.5%)
Upper limb lymphedema—27	20 (18.0%)
Nine hole peg test	19 (17.1%)
Lymphoedema functioning disability and health questionnaire	19 (17.1%)
Purdue pegboard	15 (13.5%)
Bioelectric impedance analysis	15 (13.5%)
Shoulder pain and disability index	11 (9.9%)
Volumeter/water displacement	10 (9.0%)
Functional assessment of cancer therapy—breast + 4	10 (9.0%)
Perometry	5 (4.5%)
Ultrasonography	3 (2.7%)
Myoton	3 (2.7%)
Tonometer	2 (1.8%)
Skinfibrometer	2 (1.8%)

The data for this table is from the following study: Doubblestein et al. [13]. Permission granted by chief editor of Rehabilitation Oncology and principal author

*DASH* disabilities of the arm, shoulder, and hand

### Subjects

Purposive recruitment of a heterogeneous group of qualified content experts who would have a summative understanding of BCRL was vital for this supplemental survey. A content expert was defined as a professional who had 5 or more years managing and/or researching BCRL which was the minimum inclusion criteria. Snowball sampling was instituted with primary survey disseminations through (1) SMG and SAG colleague experts, (2) interrelated conferences, and (3) listserv through the Lymphology Association of North America (LANA). Respondents were excluded if they (1) did not provide consent, (2) practiced outside of the United States or Canada, (3) had less than 5 years of experience with BCRL, or (4) survey completion included only demographic data.

The study received exempt status from the A.T. Still University—Arizona Institutional Review board. After giving written consent, the participants completed the first online survey, which was available for 30 days. Email addresses were gathered from participants who completed the first survey and subsequently received the second survey 6 weeks later which was available for 30 days. The second survey was anonymous to encourage engagement. Reminder emails were sent every 2 weeks to encourage participation. Respondents who completed both surveys received a summary of findings and a brief conclusive demographic survey.

### Data analysis

Data were analyzed using IBM SPSS Version 29 (Armonk, New York). Participants were examined to understand their demographic and practice characteristics (Table 2) and are presented as counts (*n*), means ± standard deviations, and frequencies (%). To provide a richer source of data toward understanding the respondent’s preferences, the survey allowed respondents to choose more than one choice for OM recommendations and feasibility settings. The multiple response feature of SPSS was used to assess the percent of cases and are presented as counts (*n*) and frequencies (%). Considering the consensus criteria, the SMG examined the Core Outcome Measures in Effectiveness Trials (COMET) handbook for guidance and determined to avoid criteria that was too accommodating so as not to have a long list of options, but also not too stringent that would categorically exclude data [19]. The SMG established an a priori consensus threshold of 70% agreement from respondents to appoint OMs for each OD, and ≥ 50% for examining feasibility of the OMs in various settings.

**Table 2:** Characteristics of respondents (*n* = 33)

Profession (*n* = 27)	*n* (%)
Physical therapist	15 (55.56)
Occupational therapist	7 (25.92)
Physician	2 (7.41)
Researcher	2 (7.41)
Advanced practice nurse	1 (3.70)
CLT trained with 135 CE hours (*n* = 27)	25 (92.59)
LANA credentialed CLT (*n* = 27)	20 (74.07)

	Mean ± SD
Years practicing profession (*n* = 26)	24.33 ± 10.40
Years as CLT (*n* = 23)	14.33 ± 6.91
Years managing/researching BCRL (*n* = 23)	15.13 ± 7.67

*CLT* certified lymphedema therapist, *LANA* Lymphology Association of North America, *SD* standard deviation

## Results

### Participants

Respondents (*n* = 33) completed the supplemental survey, of whom 27 completed the demographic survey including PT (*n* = 15), OT (*n* = 7), Physician (*n* = 2), BCRL Researcher (*n* = 2), and Advanced Practice Nurse (*n* = 1). These respondents had an average of 15.13 ± 7.67 years either managing and/or researching BCRL. A majority of these respondents were CLTs (*n* = 25) and were certified through LANA (*n* = 20). Further characteristics are presented in Table 2.

### Outcome measures to assess body structures and functions

The International Society of Lymphology (ISL) classification method was highly recommended for use to assess the progression of BCRL (*n* = 25, 80.6%); however, the Upper Extremity Lymphedema Index (*n* = 16, 51.6%) and Indocyanine Green (ICG) lymphography (*n* = 15, 48.4%) had a significant margin of difference (> 20%) from other measures. Both active and passive range of motion using a goniometer were recommended OMs to assess joint function (*n* = 31, 100% and *n* = 27, 87.1% respectively). Two measures were highly recommended to measure flexibility including assessment of pectoralis major length (*n* = 25, 92.6%) and stiffness of the glenohumeral joint (*n* = 25, 92.6%). Measures of strength included hand grip dynamometry (*n* = 21, 70%) and MMT (*n* = 26, 86.7%). Volume was championed by circumferential measurements converted to volume (*n* = 31, 93.9%) while tissue consistency methodology was equally qualified across (1) pitting edema test by palpation, (2) tissue texture test by palpation, and (3) axillary web syndrome by palpation (all *n* = 31, 96.9%). The numeric pain rating scale (*n* = 28, 93.3%) and visual analog scale (*n* = 26, 86.7%) were recommended OMs for pain. Light touch sensation (e.g. cotton ball, finger, brush) was the only recommended OM to assess sensation. Pioneering OMs that were highly recommended included bioelectrical impedance analysis for tissue water content (*n* = 26, 96.3%) and ultrasonography for tissue consistency (*n* = 8, 72.7%). Further information regarding recommended OMs to assess body structures and functions ODs are listed in Table 3.

**Table 3:** Recommended outcome measures (*n* = 33)

Progression or reduction of lymphedema	*n*	% of respondents
ISL stages^a^	25	80.6%
UELI	16	51.6%
ICG lymphography	15	48.4%
MRL	6	19.4%
CTCAE	4	12.9%
Lymphoscintigraphy	2	6.5%
Joint function		
Goniometry—AROM^a^	31	100.0%
Goniometry—PROM^a^	27	87.1%
Dynamic motion assessment of scapula	19	61.3%
Flexibility		
Pectoralis major length^a^	25	92.6%
Stiffness of glenohumeral joint^a^	25	92.6%
Pectoralis minor length	18	66.7%
Strength		
Manual muscle test^a^	26	86.7%
Hand grip dynamometry^a^	21	70.0%
Hand held dynamometry	18	60.0%
Pinch dynamometry	12	40.0%
Volume		
Circumference—converted to volume^a^	31	93.9%
Perometry	19	57.6%
Circumferential measurements	17	51.5%
Water displacement	8	24.2%
Pain		
Numeric pain rating scale^a^	28	93.3%
Visual analog scale^a^	26	86.7%
Sensation		
Light touch^a^	22	75.9%
Monofilament	20	69.0%
Sharp-dull discrimination	15	51.7%
Two-point discrimination	11	37.9%
Body composition		
Body weight^a^	24	88.9%
Body mass index^a^	22	81.5%
Tissue consistency		
Pitting edema test—palpation^a^	31	96.9%
Tissue texture—palpation^a^	31	96.9%
Axillary web syndrome^a^	31	96.9%
HRQOL		
LLIS^a^	27	90.0%
LYMQOL^a^	22	73.3%
Lymph-ICF	16	53.3%
ULL-27	13	43.3%
FACT-B	12	40.0%
Upper quadrant function		
* Quick*DASH^a^	23	82.1%
DASH^a^	22	78.6%
SPADI	17	60.7%
Fatigue		
Brief fatigue inventory^a^	22	84.6%
Visual analog scale—fatigue^a^	21	80.8%
Mobility and balance		
Timed up and go^a^	21	80.8%
5 × Sit to stand^a^	19	73.1%
Functional reach test	18	69.2%
6 Minute walk test	17	65.4%
Berg balance scale	16	61.5%
Upper extremity activity and motor control		
9-Hole peg test^a^	10	90.9%
Purdue pegboard	7	63.9%
Tissue water content		
Bioelectrical impedance analysis^a^	26	96.3%
Tissue dielectric constant	5	18.5%
Hi-tech tissue consistency		
Ultrasonography^a^	8	72.7%
Tonometry	7	63.6%
Skinfibrometer	3	27.3%
Myoton	2	18.2%

*ISL* International Society of Lymphology, *CTCAE* common terminology criteria of adverse events, *UELI* upper extremity lymphedema index, *ICG* indocyanine green, *MRL* magnetic resonance lymphangiography, *PROM* passive range of motion, *AROM* active range of motion, *Lymph-ICF* lymphedema functioning, disability and health, *FACT-B* functional assessment of cancer therapy—breast, *LLIS* lymphedema life impact scale, *LYMQOL* lymphedema quality of life, *ULL-27* upper limb lymphedema-27, *DASH* disability of arm, shoulder, and hand questionnaire, *SPADI* shoulder pain and disability index

^a^Met the minimum consensus threshold of 70%

### Outcome measures to assess activities and participation

The Lymphedema Life Impact Scale (LLIS) was highly recommended to assess patient-reported HRQOL (*n* = 27, 90%) and the Lymphedema Quality of Life (LYMQOL) was also recommended (*n* = 22, 73.3%). The Disability of Arm, Shoulder, and Hand questionnaire (DASH) (*n* = 22, 78.6%) and the *Quick*DASH (*n* = 23, 82.1%) were highly recommended OMs to assess upper quadrant function. Two recommended OMs for fatigue included the patient-reported Brief Fatigue Inventory (*n* = 22, 84.6%) and using a visual analog scale (*n* = 21, 80.8%). Highly recommended mobility and balance OMs included the Timed Up and GO (TUG) (*n* = 21, 80.8%) and the 5x’s Sit to Stand (5xSTS) (*n* = 19, 73.1%). The 9-hole peg test (*n* = 10, 90.9%) was the singular highly recommended OM to assess upper extremity activity and motor control. Further information regarding recommended OMs to assess activities and participation ODs are listed Table 3.

### Feasibility of highly recommended outcome measures

The ISL stages for lymphedema classification was feasible in most outpatient (*n* = 31, 93.9%), inpatient (*n* = 19, 57.6%), and research settings (*n* = 17, 51.5%). While goniometric measurements of active and passive range of motion were exceedingly feasible in most outpatient settings (*n* = 33, 100%), they were also recommended in inpatient and research settings (> 60%). Pectoralis major length measures did not meet consensus threshold for feasibility in most inpatient settings (*n* = 15, 45.5%); however, measures of stiffness of the glenohumeral joint met threshold for all settings. MMT and hand grip dynamometer were considered feasible mainly in outpatient settings (*n* = 29, 87.9% and *n* = 30, 90.9% respectively), but hand grip dynamometer did not meet consensus threshold to be feasible in most inpatient settings (*n* = 16, 48.5%). Circumferential measurements converted to volume, numeric pain scale and VAS, light touch measures, and the tissue consistency tests were considered feasible in most inpatient and research settings, but > 97% of respondents overwhelmingly considered them feasible in most outpatient settings. The self-report quality of life measures did not meet the feasibility consensus threshold for inpatient settings; LLIS (*n* = 14, 43.8%) and LYMQOL (*n* = 10, 31.3%). In fact, none of the patient-reported OMs met the consensus threshold for feasibility in most inpatient settings, including the DASH, *Quick*DASH, and Brief Fatigue Inventory. The TUG and 5xSTS assessments were feasible in most outpatient, inpatient, and research settings. While the 9-Hole Peg Test met threshold for feasibility in outpatient settings (*n* = 17, 51.5%), 48.5% (*n* = 16) of respondents reported that they had no experience with this OM.

We examined the feasibility of more high-tech OMs and discovered that while bioelectrical impedance analysis was a recommended instrument (*n* = 26, 96.3%), it was considered feasible in only outpatient (*n* = 20, 64.5%) and research settings (*n* = 21, 67.7%). Ultrasonography did not meet the feasibility consensus threshold for any setting. Additional information on the feasibility of OMs can be found in Table 4.

**Table 4:** Feasibility of outcome measures in various settings (*n* = 33)

Outpatient settings	*n*	% of respondents
Goniometry—PROM^a^	33	100.0%
Goniometry—AROM^a^	33	100.0%
Circumference—converted to volume^a^	33	100.0%
Circumferential measurements^a^	33	100.0%
Numeric pain rating scale^a^	32	100.0%
Visual analog scale^a^	32	100.0%
Pitting edema test—palpation^a^	33	100.0%
Tissue texture—palpation^a^	33	100.0%
Axillary web syndrome^a^	33	100.0%
Light touch^a^	32	97.0%
ISL stages^a^	31	93.9%
Body weight^a^	31	93.9%
Stiffness of glenohumeral joint^a^	30	90.9%
Manual muscle testing^a^	30	90.9%
Body mass index^a^	30	90.9%
Pectoralis major length^a^	29	87.9%
Hand grip dynamometry^a^	29	87.9%
Visual analog scale—fatigue^a^	29	87.9%
Timed up and go^a^	29	87.9%
5-Times sit to stand^a^	29	87.9%
LLIS^a^	27	84.4%
DASH^a^	28	84.8%
*Quick*DASH^a^	28	84.8%
LYMQOL^a^	25	78.1%
Brief fatigue inventory^a^	25	75.8%
Bioelectrical impedance analysis^a^	20	64.5%
9-Hole peg test^a^	17	51.5%
Ultrasonography	9	27.3%
Inpatient settings		
Body weight^a^	22	66.7%
Pitting edema test—palpation^a^	21	63.6%
Tissue texture—palpation^a^	21	63.6%
Axillary web syndrome^a^	21	63.6%
Numeric pain rating scale^a^	20	62.5%
Visual analog scale^a^	20	62.5%
Goniometry—PROM^a^	20	60.6%
Goniometry—AROM^a^	20	60.6%
Circumference—converted to volume^a^	20	60.6%
Circumferential measurements^a^	20	60.6%
Light touch^a^	20	60.6%
Body mass index^a^	20	60.6%
5-Times sit to stand^a^	20	60.6%
ISL^a^	19	57.6%
Timed up and go^a^	19	57.6%
Stiffness of glenohumeral joint^a^	18	54.5%
Manual muscle testing^a^	18	54.5%
Visual analog scale—fatigue^a^	17	51.5%
Hand grip dynamometry	16	48.5%
Pectoralis major length	15	45.5%
DASH	15	45.5%
LLIS	14	43.8%
* Quick*DASH	14	42.4%
Brief fatigue inventory	12	36.4%
LYMQOL	10	31.3%
Ultrasonography	9	27.3%
Bioelectrical impedance analysis	7	22.6%
9-Hole peg test	6	18.2%
Research settings		
Body mass index^a^	24	72.7%
Bioelectrical impedance analysis^a^	21	67.7%
Light touch^a^	22	66.7%
Body weight^a^	22	66.7%
Timed up and go^a^	22	66.7%
5-Times sit to stand^a^	22	66.7%
Goniometry—PROM^a^	21	63.6%
Goniometry—AROM^a^	21	63.6%
Pitting edema test—palpation^a^	21	63.6%
Tissue texture—palpation^a^	21	63.6%
Axillary web syndrome^a^	21	63.6%
Numeric pain rating scale^a^	20	62.5%
Visual analog scale^a^	20	62.5%
Pectoralis major length^a^	20	60.6%
Stiffness of glenohumeral joint^a^	20	60.6%
Hand grip dynamometry^a^	20	60.6%
Circumference—converted to volume^a^	20	60.6%
Circumferential measurements^a^	20	60.6%
DASH^a^	20	60.6%
Visual analog scale—fatigue^a^	19	57.6%
LLIS^a^	18	56.3%
Manual muscle testing^a^	18	54.5%
* Quick*DASH^a^	18	54.5%
ISL stages^a^	17	51.5%
Brief fatigue inventory	16	48.5%
LYMQOL	14	43.8%
Ultrasonography	13	39.4%
9-Hole peg test	10	30.3%

*PROM* passive range of motion*, AROM* active range of motion, *ISL* international society of lymphology, *CTCAE* common terminology criteria of adverse events, *LLIS* lymphedema life impact scale, *DASH* disability of arm, shoulder, and hand questionnaire, *LYMQOL* lymphedema quality of life

^a^Met the minimum consensus threshold of 50%

## Discussion

Consistently measuring ODs using OMs with good psychometrics helps to demonstrate therapeutic progress, document efficacy of interventions, and describe the burden of BCRL. Without the use of valid and reliable OMs to obtain objective measures there are limitations in identification of comorbidities, transfer of care, and guidance for clinical reasoning on interventions that may be detrimental for continuum of care.

### Highly recommended outcome measures that assess body functions and structures outcome domains

Clinical practice guidelines for BCRL [21, 22] recommend using circumferential measurements for the conversion to volume which aligns with what interdisciplinary experts in this study highly recommend. However, this OM may not capture the small-scale changes during the subclinical/surveillance phase for the detection of the BCRL. Current recommendations during this phase are to assess tissue water content using either tissue dielectric constant or bioelectrical impedance analysis [23]. Bioelectrical impedance analysis was highly recommended and was considered feasible for both outpatient and research settings. However, cost for such hi-tech devices may be prohibitive for non-hospital-based outpatient clinics or researchers without funding. Improving the feasibility for providers limited by such financial constraints to attain such devices is worthy of further research and goodwill.

Some of the OMs highly recommended in this study are limited by their psychometric properties. For instance, (1) dynamic motion of the scapula, (2) stiffness of the glenohumeral joint, (3) MMT, (4) pitting edema test, and (5) tissue texture by palpation are OMs assessed and interpreted subjectively by the practitioner and may be deficient in standards of reliability, especially inter-rater reliability. For example, Bittmann et al. [24] found that the reliability for the MMT was insufficient with experienced testers demonstrating inter-tester differences and low intra-rater reproducibility. Furthermore, the MMT was not recommended for clinical examination and outcomes assessment with breast cancer patients due to insufficient information on individuals with or post-cancer, whereas hand-held dynamometry is recommended for clinical practice [15]. Grip dynamometry, a reliable (ICC 2,1 = 0.96) outcome instrument, may be used as an alternative to MMT to assess not only the force of gripping but also the functional integrity of the upper limb [25]. The pitting edema test is a semi-quantitative test but lacks reproducibility with Kappa ranging 0.17 to 0.46 for the dorsum of the foot and inter-rater variability of pressures administered [26]. This measure of tissue consistency has been vetted by systematic analysis, but was not recommended for use with BCRL due to absence of diagnostic accuracy [21]. Assessment of axillary web syndrome has been established, but its reliability has yet to be evaluated [27, 28]. Expert respondents highly recommended ultrasonography as an OM and it is also recommended in the BCRL diagnosis clinical practice guideline to assess underlying tissue changes for Stage III BCRL [21]. However, this OM did not meet consensus threshold for its feasibility in outpatient, inpatient, and research setting, despite the availability of portable units. As tissue consistency is a component of the ISL staging of BCRL, there is a need for a more objective and reliable measure that is also feasible in multiple settings. Body weight and BMI met the consensus threshold for body composition and are also recommended by the Dutch Society of Dermatology [29].

### Highly recommended outcome measures that assess activities and participation outcome domains

Studies have attested to impaired physical and mental health throughout the continuum of BCRL in BCS [30–32]. In this study and according to the study by Doubblestein et al. [13], the LLIS is strongly recommended as the patient-reported HRQOL tool and in this study was marginally followed by the LYMQOL and Lymph-ICF. While the LLIS is a validated OM, a recent systematic review using COSMIN analysis reported short-comings in its development [33]. In summary, Beelen et al. suggests that there are currently no patient-reported HRQOL tools that have adequate development and have met methodological standards, but gives consideration to the Upper Limb Lymphedema-27 as a sounder tool, which was not highly recommended in this study (*n* = 13, 43.3%). A possible reason the LLIS and LYMQOL are highly recommended could be that they are easily accessible online and in the literature compared to other OMs. Although the Upper Limb Lymphedema-27 may be considered a sounder tool, it is difficult to find the questionnaire. Lack of access to OMs may affect the recommended use, especially in the clinical setting since many clinicians do not have the time or resources to find questionnaires that are not easily accessible or have a cost associated with their use. Another patient-reported outcome measure tool that was highly recommended even more than its predecessor was the *Quick*Dash. This instrument has been recommended by the American Physical Therapy—Breast Cancer EDGE Task Force and has been determined to be a valid and reliable measure for breast cancer survivors [34], but not specifically for BCRL.

Many OMs that assess activities and participation are not used by a majority of CLTs according to the supplemental digital content provided by Doubblestein et al. [13]. This is rather unfortunate, because it is through these measures that we can objectively understand the comorbidities that directly influence the HRQOL of BCS with BCRL. OMs that assess fatigue were rarely used by CLTs with their frequency of non-use ranging from 45 to 86.5% [13]. OMs assessing mobility and balance were not used by 45 to 89.2% of CLTs and OMs that measured upper extremity and motor control were not used by 69.4 to 87.4% of CLTs. While ODs and their related OMs are used more frequently to measure body structures and functions than for activities and participation [13], there were some highly recommended OMs in this study for mobility and balance and upper extremity activity and motor control. Balance disturbances have been noted in BCS and with BCS with BCRL [35, 36]. The TUG was highly recommended (*n* = 21, 80.8%) by experts in this study, is a valid and reliable OM, has been used in clinical trials with BCS with BCLR [36], and recommended by the APTA Academy of Oncologic Physical Therapy EDGE task force for adult cancer survivors [37, 38]. According to expert participants, the TUG is also feasible in most outpatient, inpatient, and research settings. The 9-Hole peg test was highly recommended (*n* = 10, 90.9%) to measure activity and motor control of the upper extremity with individuals with BCRL. While the 9-Hole Peg test has proven to be valid and reliable with Parkinson’s disease [39, 40], there is a gap in literature for its use in clinical trials for BCS and BCRL. The feasibility to use this instrument was rather low and met consensus threshold for only most outpatient settings. This may be reflective of the 48.5% of respondents reporting that they had no experience with its use.

### Strengths and limitations

The methodology for the development of the survey was a strength in that both a study management and advisory group made up of various healthcare disciplines ensured that the surveys captured the proper content. Respondents were well informed of the purpose of the study and descriptions of all survey content were available via an embedded hover feature or link to a webpage.

Some limitations need to be considered. First, neither the SMG nor SAG included a patient which may have altered the inclusion of OMs in the survey. Second, we restricted the study to the United States and Canada, therefore the recommended OMs are not intended to be international guidelines. Third, our intention was to capture BCRL OMs encompassing all potential affected areas (i.e. arm, trunk, and breast), but breast- and trunk-specific patient-reported OMs were missing from this study. The lack of these OMs is likely reflective of the challenges and lack of evidence and understanding by professionals to address lymphedema specific to breast and trunk.

## Conclusion

An integral component of a standardized core set of ODs are OMs used to measure the COS. This study identified 27 standardized OMs with a few ODs having 2–3 highly recommended OMs for proper measurement. Many of the OMs have been vetted through clinical trials with BCS with BCRL. Narrowing the choices of OMs to 27 that are highly recommended by BCRL experts may reduce selective reporting, inconsistency in clinical use, and variability of reporting across interdisciplinary healthcare fields which manage or research BCRL.

### Supplementary Information

Below is the link to the electronic supplementary material.

Supplementary file1 (PDF 683 KB)
