# Supplementary material for: Development of a core set of outcome measures to be applied toward breast cancer-related lymphedema core outcome domains
Source: Breast Cancer Res Treat. 2024 Mar 22;205(3):439–49. doi: 10.1007/s10549-024-07298-7 (PMC11101581; doi:10.1007/s10549-024-07298-7)
Supplement: Supplementary file 1 — Supplementary file1 (PDF 683 KB) [file 10549_2024_7298_MOESM1_ESM.pdf]

Supplemental Information A
COS on BCRL Second Survey

Q1 **Consent to Participate** Thank you for participating in the **first survey** of the Delphi study to develop a Core Outcome Set for Breast Cancer-Related Lymphedema. The preliminary results of the first survey can be found HERE.

In this **second survey**, we are interested in knowing what outcome measures you would incorporate into your assessment of a patient with breast cancer-related lymphedema, considering a work environment that is **NOT CONSTRAINED** in time or resources. In addition, we are investigating what instruments you would highly recommend using to measure specific outcome measures and their related feasibility in clinical settings and research settings. The instruments that are listed are not exhaustive. They have been purposefully narrowed down and taken from 92 outcome measures that have been previously investigated. The chosen instruments represent the most frequently used (50 - 100%) instruments by Certified Lymphedema Therapists to measure the outcomes. For further information, please read the following article: Use of Outcome Measures.

This survey will take approximately 20 minutes to complete. **Keywords have a hover-over feature** that will reveal their definitions. **Additional background information can be found** HERE**.** The survey software has been set so that your email address is retained alongside your survey responses. Once data has been gathered, the email addresses will be codified for anonymity. Participation in this study is completely voluntary. Should you decide to participate now, you may change your mind and stop at any time by simply exiting the survey. We expect to publish the results of this study but will not include any information that would identify you. **If you have questions about this survey, you can contact**

Principal Investigator: David Doubblestein, PT, PhD
Email: daviddoubblestein@atsu.edu
Phone: 231-629-7109
Co-Investigator: Jane Armer, RN, PhD
Email: armerj@missouri.edu

As part of their review, A.T. Still University Institutional Review Board has determined that this study is no more than minimal risk and exempt from ongoing IRB oversight. Informed Consent:
By clicking on "Yes, I agree to participate," you are consenting to participate in this survey.

If you do not wish to participate, select "No, I do not wish to participate" to exit.

- ○ Yes, I agree to participate (1)
- ○ No, I do not wish to participate (2)

Skip To: End of Survey If Consent to Participate Thank you for participating in the first survey of the Delphi study to d... = No, I do not wish to participate

Q2 This section of the survey focuses on outcome measures used to identify Body Structure and Function Impairments along the continuum of breast cancer-related lymphedema (pre-surgical, post-surgical, subclinical/surveillance, acute, and chronic). These outcome measures have been established by the APTA Academy of Oncological Physical Therapy and the Dutch Lymphedema Guidelines. For further information, please read the following article: Use of Outcome Measures. Please answer to your best ability. For best navigation through the survey, we recommend that you use a computer, laptop, or tablet and avoid using a cellular device.

Q3 Given ample time and resources, which of the following outcome measures would you **include** in your assessment of a patient during the **PRE-SURGICAL** phase on the continuum of care for breast cancer-related lymphedema? *Please move the outcome measure to the appropriate box (i.e. click-drag, and drop in the box). Do not be concerned about ranking the measures*. Click for descriptions.

Include for this phase	I am not trained to assess	NOT include for this phase
______ Joint Function (1)	______ Joint Function (1)	______ Joint Function (1)
______ Flexibility (2)	______ Flexibility (2)	______ Flexibility (2)
______ Strength (3)	______ Strength (3)	______ Strength (3)
______ Volume (4)	______ Volume (4)	______ Volume (4)
______ Pain (5)	______ Pain (5)	______ Pain (5)
______ Sensation (6)	______ Sensation (6)	______ Sensation (6)
______ Tissue consistency (7)	______ Tissue consistency (7)	______ Tissue consistency (7)
______ Body composition (8)	______ Body composition (8)	______ Body composition (8)
______ Stages of lymphedema (9)	______ Stages of lymphedema (9)	______ Stages of lymphedema (9)

Q4 Given ample time and resources, which of the following outcome measures would you **include** in your assessment of a patient during the **POST-SURGICAL** phase on the continuum of care for breast cancer-related lymphedema? *Please move the outcome measure to the appropriate box (i.e. click-drag, and drop in the box). Do not be concerned about ranking the measures*. Click for descriptions.

Include for this phase	I am not trained to assess	NOT include for this phase
______ Joint Function (1)	______ Joint Function (1)	______ Joint Function (1)
______ Flexibility (2)	______ Flexibility (2)	______ Flexibility (2)
______ Strength (3)	______ Strength (3)	______ Strength (3)
______ Volume (4)	______ Volume (4)	______ Volume (4)
______ Pain (5)	______ Pain (5)	______ Pain (5)
______ Sensation (6)	______ Sensation (6)	______ Sensation (6)
______ Tissue consistency (7)	______ Tissue consistency (7)	______ Tissue consistency (7)
______ Body composition (8)	______ Body composition (8)	______ Body composition (8)
______ Stages of lymphedema (9)	______ Stages of lymphedema (9)	______ Stages of lymphedema (9)

Q5 Given ample time and resources, which of the following outcome measures would you **include** in your assessment of a patient during the **SUBCLINICAL LYMPHEDEMA** phase on the continuum of care for breast cancer-related lymphedema? *Please move the outcome measure to the appropriate box (i.e. click-drag, and drop in the box). Do not be concerned about ranking the measures*. Click for descriptions.

Include for this phase	I am not trained to assess	NOT include for this phase
______ Joint Function (1)	______ Joint Function (1)	______ Joint Function (1)
______ Flexibility (2)	______ Flexibility (2)	______ Flexibility (2)
______ Strength (3)	______ Strength (3)	______ Strength (3)
______ Volume (4)	______ Volume (4)	______ Volume (4)
______ Pain (5)	______ Pain (5)	______ Pain (5)
______ Sensation (6)	______ Sensation (6)	______ Sensation (6)
______ Tissue consistency (7)	______ Tissue consistency (7)	______ Tissue consistency (7)
______ Body composition (8)	______ Body composition (8)	______ Body composition (8)

Q6 Given ample time and resources, which of the following outcome measures would you **include** in your assessment of a patient during the **ACUTE LYMPHEDEMA** phase on the continuum of care for breast cancer-related lymphedema? *Please move the outcome measure to the appropriate box (i.e. click-drag, and drop in the box). Do not be concerned about ranking the measures*. Click for descriptions.

Include for this phase	I am not trained to assess	NOT include for this phase
______ Joint Function (1)	______ Joint Function (1)	______ Joint Function (1)
______ Flexibility (2)	______ Flexibility (2)	______ Flexibility (2)
______ Strength (3)	______ Strength (3)	______ Strength (3)
______ Volume (4)	______ Volume (4)	______ Volume (4)
______ Pain (5)	______ Pain (5)	______ Pain (5)
______ Sensation (6)	______ Sensation (6)	______ Sensation (6)
______ Tissue consistency (7)	______ Tissue consistency (7)	______ Tissue consistency (7)
______ Body composition (8)	______ Body composition (8)	______ Body composition (8)

Q7 Given ample time and resources, which of the following outcome measures would you **include** in your assessment of a patient during the **CHRONIC LYMPHEDEMA** phase on the continuum of care for breast cancer-related lymphedema? *Please move the outcome measure to the appropriate box (i.e. click-drag, and drop in the box). Do not be concerned about ranking the measures*. Click for descriptions.

Include for this phase	I am not trained to assess	NOT include for this phase
______ Joint Function (1)	______ Joint Function (1)	______ Joint Function (1)
______ Flexibility (2)	______ Flexibility (2)	______ Flexibility (2)

______ Strength (3)	______ Strength (3)	______ Strength (3)
______ Volume (4)	______ Volume (4)	______ Volume (4)
______ Pain (5)	______ Pain (5)	______ Pain (5)
______ Sensation (6)	______ Sensation (6)	______ Sensation (6)
______ Tissue consistency (7)	______ Tissue consistency (7)	______ Tissue consistency (7)
______ Body composition (8)	______ Body composition (8)	______ Body composition (8)

Q8 This section of the survey focuses on outcome measures used to identify Activity Limitations and Participation Restrictions along the continuum of breast cancer-related lymphedema (pre-surgical, post-surgical, subclinical/surveillance, acute, and chronic). These outcome measures have been established by the APTA Academy of Oncological Physical Therapy and the Dutch Lymphedema Guidelines. For further information, please read the following article: Use of Outcome Measures. Please answer to your best ability.

Q9 Given ample time and resources, which of the following outcome measures would you **include** in your assessment of a patient during the **PRE-SURGICAL** phase on the continuum of care for breast cancer-related lymphedema? *Please move the outcome measure to the appropriate box (i.e. click-drag, and drop in the box). Do not be concerned about ranking the measures*. Click for descriptions.

Include for this phase	I am not trained to assess	NOT include for this phase
______ Patient-reported Health-related quality of life (1)	______ Patient-reported Health-related quality of life (1)	______ Patient-reported Health-related quality of life (1)
______ Patient-reported upper quadrant function (2)	______ Patient-reported upper quadrant function (2)	______ Patient-reported upper quadrant function (2)
______ Patient-reported fatigue (3)	______ Patient-reported fatigue (3)	______ Patient-reported fatigue (3)
______ Mobility and balance (4)	______ Mobility and balance (4)	______ Mobility and balance (4)
______ Upper extremity activity and motor control (5)	______ Upper extremity activity and motor control (5)	______ Upper extremity activity and motor control (5)

Q10 Given ample time and resources, which of the following outcome measures would you **include** in your assessment of a patient during the **POST-SURGICAL** phase on the continuum of care for breast cancer-related lymphedema? *Please move the outcome measure to the appropriate box (i.e. click-drag, and drop in the box). Do not be concerned about ranking the measures*. Click for descriptions.

Include for this phase	I am not trained to assess	NOT include for this phase
______ Patient-reported Health-related quality of life (1)	______ Patient-reported Health-related quality of life (1)	______ Patient-reported Health-related quality of life (1)
______ Patient-reported upper quadrant function (2)	______ Patient-reported upper quadrant function (2)	______ Patient-reported upper quadrant function (2)
______ Patient-reported fatigue (3)	______ Patient-reported fatigue (3)	______ Patient-reported fatigue (3)
______ Mobility and balance (4)	______ Mobility and balance (4)	______ Mobility and balance (4)

______ Upper extremity activity and motor control (5)	______ Upper extremity activity and motor control (5)	______ Upper extremity activity and motor control (5)

Q11 Given ample time and resources, which of the following outcome measures would you **include** in your assessment of a patient during the **SUBCLINICAL LYMPHEDEMA** phase on the continuum of care for breast cancer-related lymphedema? *Please move the outcome measure to the appropriate box (i.e. click-drag, and drop in the box). Do not be concerned about ranking the measures*. Click for descriptions.

Include for this phase	I am not trained to assess	NOT include for this phase
______ Patient-reported Health-related quality of life (1)	______ Patient-reported Health-related quality of life (1)	______ Patient-reported Health-related quality of life (1)
______ Patient-reported upper quadrant function (2)	______ Patient-reported upper quadrant function (2)	______ Patient-reported upper quadrant function (2)
______ Patient-reported fatigue (3)	______ Patient-reported fatigue (3)	______ Patient-reported fatigue (3)
______ Mobility and balance (4)	______ Mobility and balance (4)	______ Mobility and balance (4)
______ Upper extremity activity and motor control (5)	______ Upper extremity activity and motor control (5)	______ Upper extremity activity and motor control (5)

Q12 Given ample time and resources, which of the following outcome measures would you **include** in your assessment of a patient during the **ACUTE LYMPHEDEMA** phase on the continuum of care for breast cancer-related lymphedema? *Please move the outcome measure to the appropriate box (i.e. click-drag, and drop in the box). Do not be concerned about ranking the measures*. Click for descriptions.

Include for this phase	I am not trained to assess	NOT include for this phase
______ Patient-reported Health-related quality of life (1)	______ Patient-reported Health-related quality of life (1)	______ Patient-reported Health-related quality of life (1)
______ Patient-reported upper quadrant function (2)	______ Patient-reported upper quadrant function (2)	______ Patient-reported upper quadrant function (2)
______ Patient-reported fatigue (3)	______ Patient-reported fatigue (3)	______ Patient-reported fatigue (3)
______ Mobility and balance (4)	______ Mobility and balance (4)	______ Mobility and balance (4)
______ Upper extremity activity and motor control (5)	______ Upper extremity activity and motor control (5)	______ Upper extremity activity and motor control (5)

Q13 Given ample time and resources, which of the following outcome measures would you **include** in your assessment of a patient during the **CHRONIC LYMPHEDEMA** phase on the continuum of care for breast cancer-related lymphedema? *Please move the outcome measure to the appropriate box (i.e. click-drag, and drop in the box). Do not be concerned about ranking the measures*. Click for descriptions.

Include for this phase	I am not trained to assess	NOT include for this phase
______ Patient-reported Health-related quality of life (1)	______ Patient-reported Health-related quality of life (1)	______ Patient-reported Health-related quality of life (1)
______ Patient-reported upper quadrant function (2)	______ Patient-reported upper quadrant function (2)	______ Patient-reported upper quadrant function (2)

______ Patient-reported fatigue (3)

______ Mobility and balance (4)

______ Upper extremity activity and motor control (5)

______ Patient-reported fatigue (3)

______ Mobility and balance (4)

______ Upper extremity activity and motor control (5)

______ Patient-reported fatigue (3)

______ Mobility and balance (4)

______ Upper extremity activity and motor control (5)

Q14 In this section of the second survey we are investigating what instruments you would highly recommend using to measuring the listed outcome measures and their related feasibility (the state of being conveniently used) in clinical settings and research settings. The instruments listed are not exhaustive. They have been purposefully narrowed down and taken from 92 outcome measures that have been previously investigated. The following choices represent the most frequently used (50 - 100%) instruments by Certified Lymphedema Therapists to measure the outcomes. For further information, please read the following article: Use of Outcome Measures. For best navigation through the survey, we recommend that you use a computer, laptop, or tablet and avoid using a cellular device.

Q15 Which of the following outcome measure instruments would you recommend to use to measure **THE PROGRESSION OR REDUCTION OF LYMPHEDEMA** on clients with breast cancer-related lymphedema? Click here for a description of outcome measures**.**

	Highly Recommended (1)	Not as Highly Recommended (2)	Not Recommended (3)	Unfamiliar with instrument (6)
International Society of Lymphology (ISL) Stages (1)	○	○	○	○
Common Terminology Criteria of Adverse Events (CTCAE) (2)	○	○	○	○
Upper Extremity Lymphedema Index (UELI) (3)	○	○	○	○
Indocyanine Green (ICG) Lymphography (10)	○	○	○	○
Magnetic Resonance Lymphangiography (MRL) (11)	○	○	○	○
Lymphoscintigraphy (12)	○	○	○	○

Q16 Please indicate the **FEASIBILITY** of using these outcome measurement tools in the clinical and/or research setting depending on your experience. Click here for a description of outcome measures**.**

	Feasible in most outpatient settings (11)	Feasible in most inpatient settings (2)	Feasible in most research settings (6)	No experience (8)
International Society of Lymphology (ISL) Stages (1)	☐	☐	☐	☐
Common Terminology Criteria of Adverse Events (CTCAE) (2)	☐	☐	☐	☐
Upper Extremity Lymphedema Index (UELI) (3)	☐	☐	☐	☐
Indocyanine Green (ICG) Lymphography (11)	☐	☐	☐	☐
Magnetic Resonance Lymphangiography (MRL) (12)	☐	☐	☐	☐
Lymphoscintigraphy (13)	☐	☐	☐	☐

Q17 Which of the following outcome measure instruments would you recommend to use to measure **JOINT FUNCTION** on clients with breast cancer-related lymphedema? Click here for a description of outcome measures**.**

	Highly Recommended (1)	Not as Highly Recommended (2)	Not Recommended (3)	Unfamiliar with instrument (6)
Dynamic Motion Assessment of Scapula (dichotomous) (1)	○	○	○	○
Goniometry - passive range of motion of Upper Extremity (2)	○	○	○	○
Goniometry - active range of motion of Upper Extremity (3)	○	○	○	○

Q18 Please indicate the **FEASIBILITY** of using these outcome measurement tools in the clinical and/or research setting depending on your experience. Click here for a description of outcome measures**.**

	Feasible in most outpatient settings (1)	Feasible in most inpatient settings (2)	Feasible in most research settings (7)	No experience (8)
Dynamic Motion Assessment of Scapula (dichotomous) (1)	▢	▢	▢	▢
Goniometry - passive range of motion of Upper Extremity (2)	▢	▢	▢	▢
Goniometry - active range of motion of Upper Extremity (3)	▢	▢	▢	▢

Q19 Which of the following outcome measure instruments would you recommend to measure **FLEXIBILITY** on your clients with breast cancer-related lymphedema? Click here for a description of outcome measures**.**

	Highly Recommended (1)	Not as Highly Recommended (2)	Not Recommended (3)	Unfamiliar with instrument (5)
Pectoralis major length (7)	o	o	o	o
Pectoralis minor muscle length (1)	o	o	o	o
Stiffness of glenohumeral joint (3)	o	o	o	o

Q20 Please indicate the **FEASIBILITY** of using these outcome measurement tools in the clinical and/or research setting depending on your experience. Click here for a description of outcome measures**.**

	Feasible in most outpatient settings (1)	Feasible in most inpatient settings (2)	Feasible in most research settings (5)	No experience (7)
Pectoralis major length (7)	▢	▢	▢	▢
Pectoralis minor muscle length (1)	▢	▢	▢	▢
Stiffness of glenohumeral joint (3)	▢	▢	▢	▢

Q21 Which of the following outcome measure instruments would you recommend to measure **STRENGTH** on your clients with breast cancer-related lymphedema? Click here for a description of outcome measures**.**

	Highly Recommended (1)	Not as Highly Recommended (2)	Not Recommended (3)	Unfamiliar with instrument (5)
Hand Grip Dynamometry (1)	o	o	o	o
Hand Held Dynamometry (2)	o	o	o	o
Manual Muscle Testing (MMT) (3)	o	o	o	o
Pinch Dynamometry (Tip, Lateral, 3 Jaw Chuck) (5)	o	o	o	o

Q22 Please indicate the **FEASIBILITY** of using these outcome measurement tools in the clinical and/or research setting depending on your experience. Click here for a description of outcome measures**.**

	Feasible in most outpatient settings (1)	Feasible in most inpatient settings (2)	Feasible in most research settings (5)	No experience (7)
Hand Grip Dynamometry (1)	☐	☐	☐	☐
Hand Held Dynamometry (2)	☐	☐	☐	☐
Manual Muscle Testing (MMT) (3)	☐	☐	☐	☐
Pinch Dynamometry (Tip, Lateral, 3 Jaw Chuck) (5)	☐	☐	☐	☐

Q23 Which of the following outcome measure instruments would you recommend to measure **VOLUME** on your clients with breast cancer-related lymphedema? Click here for a description of outcome measures**.**

	Highly Recommended (1)	Not as Highly Recommended (2)	Not Recommended (3)	Unfamiliar with instrument (5)
Circumferential Measures - Converted to Volume (2)	○	○	○	○
Circumferential Measurements (5)	○	○	○	○
Perometry (11)	○	○	○	○
Water Displacement (12)	○	○	○	○

Q24 Please indicate the **FEASIBILITY** of using these outcome measurement tools in the clinical and/or research setting depending on your experience. Click here for a description of outcome measures**.**

	Feasible in most outpatient settings (1)	Feasible in most inpatient settings (2)	Feasible in most research settings (5)	No experience (7)
Circumferential Measures - Converted to Volume (2)	☐	☐	☐	☐
Circumferential Measurements (5)	☐	☐	☐	☐
Perometry (11)	☐	☐	☐	☐
Water Displacement (12)	☐	☐	☐	☐

Q25 Which of the following outcome measure instruments would you recommend to measure **PAIN** on your clients with breast cancer-related lymphedema? Click here for a description of outcome measures**.**

	Highly Recommended (1)	Not as Highly Recommended (2)	Not Recommended (3)	Unfamiliar with instrument (5)
Numeric Pain Rating Scale (5)	○	○	○	○
Visual Analog Scale (7)	○	○	○	○

Q26 Please indicate the **FEASIBILITY** of using these outcome measurement tools in the clinical and/or research setting depending on your experience. Click here for a description of outcome measures**.**

	Feasible in most outpatient settings (1)	Feasible in most inpatient settings (2)	Feasible in most research settings (5)	No experience (7)
Numeric Pain Rating Scale (5)	☐	☐	☐	☐
Visual Analog Scale (7)	☐	☐	☐	☐

Q27 Which of the following outcome measure instrument would you recommend to measure **SENSATION** on your clients with breast cancer-related lymphedema? Click here for a description of outcome measures**.**

	Highly Recommended (1)	Not as Highly Recommended (2)	Not Recommended (3)	Unfamiliar with instrument (5)
Light Touch (e.g. cotton ball, finger, brush) (1)	○	○	○	○
Monofilament (2)	○	○	○	○
Sharp-Dull Discrimination (3)	○	○	○	○
Two-Point Discrimination (4)	○	○	○	○

Q28 Please indicate the **FEASIBILITY** of using these outcome measurement tools in the clinical and/or research setting depending on your experience. Click here for a description of outcome measures**.**

	Feasible in most outpatient settings (1)	Feasible in most inpatient settings (2)	Feasible in most research settings (5)	No experience (7)
Light Touch (e.g. cotton ball, finger, brush) (1)	☐	☐	☐	☐
Monofilament (2)	☐	☐	☐	☐
Sharp-Dull Discrimination (3)	☐	☐	☐	☐
Two-Point Discrimination (4)	☐	☐	☐	☐

Q29 Which of the following outcome measure instruments would you recommend to measure **TISSUE CONSISTENCY** on your clients with breast cancer-related lymphedema? Click here for a description of outcome measures**.**

	Highly Recommended (1)	Not as Highly Recommended (2)	Not Recommended (3)	Unfamiliar with instrument (5)
Pitting Edema Test - Palpation (2)	○	○	○	○
Tissue Texture - Palpation (normal, soft, spongy, firm) (8)	○	○	○	○
Axillary Web Syndrome (10)	○	○	○	○

Q30 Please indicate the **FEASIBILITY** of using these outcome measurement tools in the clinical and/or research setting depending on your experience. Click here for a description of outcome measures**.**

	Feasible in most outpatient settings (1)	Feasible in most inpatient settings (2)	Feasible in most research settings (5)	No experience (7)
Pitting Edema Test - Palpation (2)	▢	▢	▢	▢
Tissue Texture - Palpation (normal, soft, spongy, firm) (8)	▢	▢	▢	▢
Axillary Web Syndrome (10)	▢	▢	▢	▢

Q31 Which of the following outcome measure instruments would you recommend to measure **BODY COMPOSITION** on your clients with breast cancer-related lymphedema? Click here for a description of outcome measures**.**

	Highly Recommended (1)	Not as Highly Recommended (2)	Not Recommended (3)	Unfamiliar with instrument (5)
Body Weight (2)	○	○	○	○
Body Mass Index (3)	○	○	○	○

Q32 Please indicate the **FEASIBILITY** of using these outcome measurement tools in the clinical and/or research setting depending on your experience. Click here for a description of outcome measures**.**

	Feasible in most outpatient settings (1)	Feasible in most inpatient settings (2)	Feasible in most research settings (5)	No experience (7)
Body Weight (2)	▢	▢	▢	▢
Body Mass Index (3)	▢	▢	▢	▢

Q33 This third section of the survey focuses on outcome measures used to identify Activities and Participation Limitations. Please answer to your best ability.

Q34 Which of the following **PATIENT-REPORTED** outcome measure instruments would you recommend to measure **HEALTH-RELATED QUALITY OF LIFE** on your clients with breast cancer-related lymphedema? Click here for a description of outcome measures**.**

	Highly Recommended (1)	Not as Highly Recommended (2)	Not Recommended (3)	Unfamiliar with instrument (5)
Lymphedema Functioning, Disability, & Health (Lymph-ICF) (14)	o	o	o	o
Functional Assessment of Cancer Therapy - Breast (FACT-B) (4)	o	o	o	o
Lymphedema Life Impact Scale (LLIS) (7)	o	o	o	o
Lymphedema Quality of Life (LYMQOL) (18)	o	o	o	o
Upper Limb Lymphedema 27 (ULL-27) (19)	o	o	o	o

Q35 Please indicate the **FEASIBILITY** of using these outcome measurement tools in the clinical and/or research setting depending on your experience. Click here for a description of outcome measures**.**

	Feasible in most outpatient settings (1)	Feasible in most inpatient settings (2)	Feasible in most research settings (5)	No experience (7)
Lymphedema Functioning, Disability, & Health (Lymph-ICF) (14)	▢	▢	▢	▢
Functional Assessment of Cancer Therapy - Breast (FACT-B) (4)	▢	▢	▢	▢
Lymphedema Life Impact Scale (LLIS) (7)	▢	▢	▢	▢
Lymphedema Quality of Life (LYMQOL) (18)	▢	▢	▢	▢
Upper Limb Lymphedema 27 (ULL-27) (19)	▢	▢	▢	▢

Q36 Which of the following **PATIENT-REPORTED** outcome measure instruments would you recommend to measure **UPPER QUADRANT FUNCTION** on your clients with breast cancer-related lymphedema? Click here for a description of outcome measures**.**

	Highly Recommended (1)	Not as Highly Recommended (2)	Not Recommended (3)	Unfamiliar with instrument (5)
Disability of Arm, Shoulder, and Hand Questionnaire (DASH) (2)	○	○	○	○
Shoulder Pain and Disability Index (SPADI) (18)	○	○	○	○
QuickDASH (10)	○	○	○	○

Q37 Please indicate the **FEASIBILITY** of using these outcome measurement tools in the clinical and/or research setting depending on your experience. Click here for a description of outcome measures**.**

	Feasible in most outpatient settings (1)	Feasible in most inpatient settings (2)	Feasible in most research settings (5)	No experience (7)
Disability of Arm, Shoulder, and Hand Questionnaire (DASH) (2)	▢	▢	▢	▢
Shoulder Pain and Disability Index (SPADI) (18)	▢	▢	▢	▢
QuickDASH (10)	▢	▢	▢	▢

Q38 Which of the following **PATIENT-REPORTED** outcome measure instruments would you recommend to measure **FATIGUE** on your clients with breast cancer-related lymphedema? Click here for a description of outcome measures**.**

	Highly Recommended (1)	Not as Highly Recommended (2)	Not Recommended (3)	Unfamiliar with Instrument (5)
Brief Fatigue Inventory (2)	○	○	○	○
Visual Analog Scale - Fatigue (12)	○	○	○	○

Q39 Please indicate the **FEASIBILITY** of using these outcome measurement tools in the clinical and/or research setting depending on your experience. Click here for a description of outcome measures**.**

	Feasible in most outpatient settings (1)	Feasible in most inpatient settings (2)	Feasible in most research settings (5)	No experience (7)
Brief Fatigue Inventory (2)	▢	▢	▢	▢
Visual Analog Scale - Fatigue (12)	▢	▢	▢	▢

Q40 Which of the following outcome measure instruments would you recommend to measure **MOBILITY AND BALANCE** on your clients with breast cancer-related lymphedema? Click here for a description of outcome measures**.**

	Highly Recommended (1)	Not as Highly Recommended (2)	Not Recommended (3)	Unfamiliar with instrument (5)
Berg Balance Scale (3)	○	○	○	○
Functional Reach Test (11)	○	○	○	○
Timed Up and Go (10)	○	○	○	○
5 - Times Sit to Stand (12)	○	○	○	○
6 - Minute Walk Test (13)	○	○	○	○

Q41 Please indicate the **FEASIBILITY** of using these outcome measurement tools in the clinical and/or research setting depending on your experience. Click here for a description of outcome measures**.**

	Feasible in most outpatient settings (1)	Feasible in most inpatient settings (2)	Feasible in most research settings (5)	No experience (7)
Berg Balance Scale (3)	▢	▢	▢	▢
Functional Reach Test (11)	▢	▢	▢	▢
Timed Up and Go (10)	▢	▢	▢	▢
5 - Times Sit to Stand (12)	▢	▢	▢	▢
6 - Minute Walk Test (13)	▢	▢	▢	▢

Q42 Which of the following outcome measure instruments would you recommend measure **UPPER EXTREMITY ACTIVITY & MOTOR CONTROL** on your clients with breast cancer-related lymphedema? Click here for a description of outcome measures**.**

	Highly Recommended (1)	Not as Highly Recommended (2)	Not Recommended (3)	Unfamiliar with instrument (5)
Purdue Pegboard (4)	○	○	○	○
9 - Hole Peg Test (6)	○	○	○	○

Q43 Please indicate the **FEASIBILITY** of using these outcome measurement tools in the clinical and/or research setting depending on your experience. Click here for a description of outcome measures**.**

	Feasible in most outpatient settings (1)	Feasible in most inpatient settings (2)	Feasible in most research settings (5)	No experience (7)
Purdue Pegboard (4)	▢	▢	▢	▢
9 - Hole Peg Test (6)	▢	▢	▢	▢

Q44 There are tools that measure tissue water content and volume which are not frequently used. However, we need further input from your expertise. Which of the following outcome measure instruments would you recommend to measure **TISSUE WATER CONTENT AND VOLUME** on your clients with breast cancer-related lymphedema? Click here for a description of outcome measures**.**

	Highly Recommended (1)	Not as Highly Recommended (2)	Not Recommended (3)	Unfamiliar with instrument (5)
Bioelectrical Impedance Analysis (4)	○	○	○	○
Tissue Dielectric Constant (12)	○	○	○	○
3D Imaging (15)	○	○	○	○

Q45 Please indicate the **FEASIBILITY** of using these outcome measurement tools in the clinical and/or research setting depending on your experience. Click here for a description of outcome measures**.**

	Feasible in most outpatient settings (1)	Feasible in most inpatient settings (2)	Feasible in most research settings (5)	No experience (7)
Bioelectrical Impedance Analysis (4)	▢	▢	▢	▢
Tissue Dielectric Constant (12)	▢	▢	▢	▢
3D Imaging (15)	▢	▢	▢	▢

Q46 There are tools not frequently used that measure tissue elasticity/stiffness which gives quantitative values for fibrosis. However, we need further input from your expertise. Which of the following outcome measure instruments would you recommend to measure **TISSUE CONSISTENCY** on your clients with breast cancer-related lymphedema? Click here for a description of outcome measures**.**

	Highly Recommended (1)	Not as Highly Recommended (2)	Not Recommended (3)	Unfamiliar with instrument (5)
Ultrasonography (10)	○	○	○	○
Myoton (11)	○	○	○	○
SkinFibrometer (13)	○	○	○	○
Tonometry (14)	○	○	○	○

Q47 Please indicate the **FEASIBILITY** of using these outcome measurement tools in the clinical and/or research setting depending on your experience. Click here for a description of outcome measures**.**

	Feasible in most outpatient settings (1)	Feasible in most inpatient settings (2)	Feasible in most research settings (5)	No experience (7)
Ultrasonography (10)	☐	☐	☐	☐
Myoton (11)	☐	☐	☐	☐
SkinFibrometer (13)	☐	☐	☐	☐
Tonometry (14)	☐	☐	☐	☐

Supplemental Information B

Outcome Measure Intrument	Description
International Society of Lymphology (ISL) Stages	A staging classification system that indicates the characteristics and severity of lymphedema progression. Stages: 0 = normal clinical presentation, but with abnormal lymph transport; 1 = early edema which improves with upper extremity elevation; 2 = pitting edema presentation and edema does not resolve with elevation; 3 = significant fibrotic tissue and/or adiposity, and skin changes. Severity: mild (<20% volume increase); moderate (20–40% volume increase); severe (>40% volume increase).
Common Terminology Criteria of Adverse Events (CTCAE)	A standard classification and severity grading scale for adverse events (abnormal clinical finding) associated to cancer therapy. Grades: 1 = trace thickening or faint discoloration; 2 = marked discoloration/leathery skin texture/papillary formation/limiting instrumental ADLs; 3 = severe symptoms/limiting self-care ADLs.
Upper Extremity Lymphedema Index (UELI)	A lymphedema index score derived by a mathematical calculation using circumferential measurements and body mass index. The index correlates with conventional clinical stages and can used to determine lymphedema severity.
Idocyanine Green (ICG) Lymphography	An imaging technique that uses water-based indocyanine green fluorescence solution which allows for quick visualization of superficial lymph flow in real-time, without radiation exposure. Images of dermal backflow (e.g. linear, splash, stardust, and diffuse patterns) correlate with progression of lymphedema.
Magnetic Resonance Lymphangiography (MRL)	An imaging technique that uses a contrast agent (gadolinium-based) to visualize and map the lymphatic vessels, and diagnose lymphedema.
Lymphoscintigraphy	An imaging technique that uses an intradermal or subcutaneous injection of a radiolabeled tracer and an imaging system using a gamma camera.
Dynamic Motion Assessment of Scapula	Also known as Scapular Dyskinesis Test (McClure). Patient performs 5 slow repetitions of shoulder flexion and then abduction with weights in hands. Dysrhythmia or winging is rated 1. Normal, 2. Subtle abnormality, 3. Obvious abnormality.
Goniometry - passive range of motion of Upper Extremity	An examiner uses a goniometer instrument to measure joint angles of a pateint which the examiner passively placed the joint in.
Goniometry - active range of motion of Upper Extremity	An examiner uses a goniometer instrument to measure joint angles being displaced actively by the pateint.
Pectoralis major length	Patient lies supine. Clavicle portion: Examiner places patients shoulder in 90 degrees of horizontal abduction and external rotation. Sternal portion: Examiner places shoulder at 120-135 degrees of abduction. Normal = upper extremity is able to lay flat on table.
Pectoralis minor muscle length	Patient lies supine with upper extremities by their sides. Examiner measure with ruler from table to posterior angle of the acromion and compares bilaterally.
Stiffness of glenohumeral joint	Patient is supine. Examiner performs passive accessory motions of the glenohumeral joint anterior/latera/posterior/inferior. Examiner compares end-feel bilaterally.
Hand Grip Dynamometry	Use of a grip dynamometer to measure isometric strength of hand grip.
Hand Held Dynamometry	Use of a hand held dynamometer to measure isometric strength of various motions of the upper extremity (flexion, abduction, external rotation) or a specific muscle strength.
Manual Muscle Testing (MMT)	Examiner uses their own internal/subjective interpretation of patient force being exerted against the forces of gravity and/or manual resistance.

Pinch Dynamometry (Tip, Lateral, 3 Jaw Chuck)	Use of a dynamometer to measure isometric strength of pinching in different ways.
Circumferential Measures - Converted to Volume	Examiner uses a tape measure to measure the circumference of the upper extremity at regular longitudinal intervals of anatomic locations. The examiner then converts these measurements into a volume measure. Also known at truncated cone volume measurement.
Circumferential Measurements	Examiner uses a measuring tape to measure the circumference of an upper extremity at selected anatomic landmarks.
Perometry	Uses an infrared light beam and light beam receiver to measure the outline of the upper extremity and then calculates the upper extremity volume.
Water Displacement	An upper extremity is submersed into a container with water which displaces the water into another container. The water is measured as volume. A volumeter instrument is typically used for displacement of water.
Numeric Pain Rating Scale	Patients circle or report a number between 0 and 10 that describes their pain intensity
Visual Analog Scale	A patient makes a mark on a straight line with endpoints labeled as "no pain" and "severe pain" to describe their pain level.
Light Touch (e.g. cotton ball, finger, brush)	A measure of surface/superficial skin tactile sensitivity/perception.
Monofilament	A monofilament is a synthetic filament depressed onto skin use for assessing the loss of protective sensation.
Sharp-Dull Discrimination	A tactile perception test where an examiner uses a dull and a sharp object in order to assess the ability discriminate between the two objects.
Two-Point Discrimination	Used to assess a patient's ability to identify two points close to each other on a small area of skin.
Pitting Edema Test - Palpation	Examiner depresses the skin with a finger and measure the depth of the indention. Examiner also records how long it takes for the skin to rebound back to its original position.
Tissue Texture - Palpation (normal, soft, spongy, firm)	Examiner depresses the skin with a finger and describes the texture of the skin subjectively as normal, soft, spongy, or firm.
Axillary Web Syndrome	Examiner palpates for the presence of Axillary Web Syndrome (AWS) (a.k.a. cording, tethering, or banding) which is a condition that occurs after surgical treatment for breast cancer. Palpable thick or thin fibrotic bands/cords develop under the skin of the armpit and may extend down to the hand.
Body Weight	The measurement of weight on the patient.
Body Mass Index	A person's weight divided by the square of their height.
Lymphedema Functioning, Disability, & Health (Lymph-ICF)	A patient-reported outcome measure on quality of life using 5 domains (physical function, mental function, general tasks/household activities, mobility activities, social life) and 28 items/questions.
Functional Assessment of Cancer Therapy - Breast (FACT-B)	A patient-reported outcome measure on quality of life using 5 domains (physical, social, emotional, functional well-being, and a breast-cancer subscale) and 37 items/questions.
Lymphedema Life Impact Scale (LLIS)	A patient-reported outcome measure on quality of life using 3 domains (function, psychosocial, physical) and 18 items/questions.
Lymphedema Quality of Life (LYMQOL)	A patient-reported outcome measure on quality of life using 4 domains (function, body image/appearance, symptoms, mood) and 24 items/questions.
Upper Limb 27 (ULL-27)	A patient-reported outcome measure on quality of life using 3 domains (physical, emotional, social) and 27 items/questions.

Disability of Arm, Shoulder, and Hand Questionnaire (DASH)	A patient-reported outcome measure that uses 30 items to measure patient symptoms and physical function related to disorders affecting the upper extremity.
Shoulder Pain and Disability Index (SPADI)	A patient-reported outcome measure that measures pain (5 questions) and limitations of functional activities (8 questions) related to disorders of the upper extremity.
QuickDASH	A shortened version of the DASH using only 11 items to measure patient symptoms and physical function related to disorders affecting the upper extremity.
Brief Fatigue Inventory	A patient-reported outcome measure that uses 9 scales to rapidly assess the fatigue severity in the past 24 hours.
Visual Analog Scale - Fatigue	A patient makes a mark on a straight line with endpoints labeled as "no fatigue" and "severe fatigue" to describe their pain level.
Berg Balance Scale	Assesses a patient's balance using 14 predetermined tasks (transfers, standing with eyes open/closed, reaching, turning around, tandem step, etc.). Each item consists has a five-point scale, with 0 indicating the lowest level of function and 4 the highest level of function.
Functional Reach Test	Assesses dynamic balance in which a patient is standing and maximally reaches forward toward a wall with an outstretched upper extremity while maintaining feet on floor. A measure of distance in centimeters is compared to a scale for risk of falls.
Timed Up and Go	A timed mobility test assessing function/fall risk/balance, where a patient rises from a chair, walks 3 meters and then turns around and walks back, and then sits down again.
5 - Times Sit to Stand	A mobility test based on the amount of time a patient is able to transfer from a seated position to standing and back for a total of five times.
6 - Minute Walk Test	A mobility test used to assess endurance in which a patient sub-maximally walks for 6 minutes and the distance achieved is compared to normative data and previous data for a progressive continuum.
Purdue Pegboard	A timed quantitative assessment used to measure gross movements of upper extremity and fingertip dexterity, where a patient manipulates small pegs, collars, and washers on a board.
9 - Hole Peg Test	A quantitative assessment used to measure finger dexterity, where a patient manipulates small pegs, into and out of holes on a board over a period of time.
Bioelectrical Impedance Analysis	Bioelectrical impedance analysis uses an electrical current that travels through the body and measures body composition based on the various tissue and fluid resistances to the electrical current. Also known as bioimpedance spectroscopy analysis.
Tissue Dielectric Constant	A device that has a probe which emits a low-power electromagnetic wave and determines the dielectric constant of the tissue. It is used for quantifying local tissue water content of lymphedema.
3D Imaging	A portable three-dimensional imaging and software system that measures upper extremity circumference and the volume of an upper extremity.
Ultrasonography	Ultrasound imaging for lymphedema has been used to quantify tissue texture (fibrosis) and subcutaneous tissue fluid.
Tonometry	Tonometers are instruments that provide an indentation of a blunt probe into the skin resulting in tissue deformation, which then gives measures proportional to the firmness/pliability of the skin. Used to assess pitting edema, fibrosis, and scar tissue.
Myoton	A type of tonometer (refer to tonometry)
SkinFibrometer	A type of tonometer (refer to tonometry)
